# Biofloc technology significantly reshapes water microbiome and improves survival rates in Japanese eel (*Anguilla japonica*)

**DOI:** 10.1128/spectrum.02206-24

**Published:** 2025-01-15

**Authors:** Jiho Yang, Hyunjun Choi, Jun Seong Park, Yehyeon Cha, Ju-Ae Hwang, Seung-Yoon Oh

**Affiliations:** 1 Gyeongnam Bio and Anti-aging Core Facility, Changwon National University 34925 , Changwon, South Korea; 2 Department of Biology and Chemistry, Changwon National University 34925 , Changwon, South Korea; 3 Advanced Aquaculture Research Center, National Institute of Fisheries Science, Changwon, South Korea; Lerner Research Institute, Cleveland, Ohio, USA

**Keywords:** aquaculture, biofloc technology (BFT), flow-through system (FTS), microbiome, Japanese eel

## Abstract

Global aquaculture production faces the challenge of biologically cycling nitrogenous waste. Biofloc technology (BFT) systems offer the potential to reduce water consumption and eliminate waste products by using beneficial microorganisms to convert waste into usable nutrients or non-toxic molecules. Unlike flow-through systems (FTS), which depend on continuous water exchange and result in higher operational costs as well as limited microbiome stability, BFT operates without the need for constant water exchange. Instead, it leverages its inherent microbiome for nutrient recycling, which can help reduce associated costs. The Japanese eel (*Anguilla japonica*), consumed as a luxury food item in China, Japan, and South Korea, has seen little research related to BFT. In this study, we observed the survival and growth rates of eels treated with BFT over 8 weeks and monitored changes in the gut and water microbiome using 16S rRNA amplicon sequencing (metabarcoding) and quantitative PCR. Our research demonstrated that BFT treatment was more advantageous for eel growth compared with FTS in terms of eel survival rate, feed intake amount, and growth rate. Both treatments did not result in water quality conditions detrimental to the growth of eels. Alpha diversity was higher in BFT water microbiome than in FTS, but no differences were observed in the guts of eels between the two treatments. While community structures of water microbiome (taxonomic composition) differed between BFT and FTS, gut microbiome did not. To identify the operational taxonomic units (OTUs) that directly influence the feed consumption rate of eels, closely linked to their growth rate, we first determined the differentially abundant residents and then highlighted the most informative OTUs. In summary, our analysis identified microbial residents potentially associated with the higher growth rates observed in BFT-treated eels, highlighting the need for further functional studies.

**IMPORTANCE:**

This study is significant as it addresses a critical gap in the application of Biofloc Technology (BFT) to Japanese eel (*Anguilla japonica*) aquaculture, a high-value species in East Asia. BFT’s potential to reduce water consumption and enhance growth through the use of beneficial microorganisms presents a sustainable solution to the challenges of nitrogenous waste management in global aquaculture. Our research provides the first comprehensive analysis of how BFT influences both the growth and microbiome composition of Japanese eels compared with traditional flow-through systems. By identifying specific microbial residents potentially linked to improved feed consumption and growth, this study opens new avenues for optimizing BFT in eel farming. The findings contribute to the broader understanding of microbial roles in aquaculture, highlighting the potential for BFT to support more sustainable and productive aquaculture practices.

## INTRODUCTION

Since the 1950s, global aquaculture production has seen significant growth, now accounting for 52% of the total weight of aquatic organisms harvested for human consumption, and it remains a crucial component of global food production (1, 2). However, intensive aquaculture has led to substantial environmental issues, such as waste accumulation and water resource depletion. Current aquaculture systems face the challenge of biologically cycling nitrogenous waste, including ammonia and its nitrification products, nitrite and nitrate (3). Common sources of ammonia influx in aquaculture include fish excreta, overfeeding, and surface aeration (4). Toxic ammonia impacts the stability of tanks and hatcheries, influencing molting, growth, oxygen consumption, and the activity of key protein enzymes in crustaceans and fish (5–7). This has led to increased global attention on the diversity of biological groups involved in nitrogen cycling and their impacts. Biofloc technology (BFT) systems offer the potential to reduce water consumption and eliminate waste products by using beneficial bacteria, microalgae, and other microorganisms to convert waste into usable nutrients or non-toxic molecules (2, 8–11). BFT systems also provide nutrient-rich water for fish growth, activating microbial communities to efficiently utilize nitrogenous wastes and enhancing nutrition through complex spatial interactions (12–16). In BFT, ammonia is metabolized through three pathways: photoautotrophic uptake by phytoplankton, chemoautotrophic transformation into less toxic nitrate, and heterotrophic conversion into consumable biomass (9, 17, 18). A flow-through system (FTS) depends on continuous water exchange to maintain water quality, which increases operational costs and limits the establishment of a stable microbiome. In contrast, BFT utilizes its internal microbiome to recycle nutrients, eliminating the need for frequent water exchange and offering a cost-effective alternative.

Microbial communities in aquaculture systems play vital roles in maintaining system health and performance (18). These microbes break down organic matter and cycle nutrients and remove toxins, essential for the growth and survival of fish and other aquatic organisms (19). Microorganisms also produce important biomolecules, such as enzymes and bioactive compounds, useful in aquaculture (20). Bacterial communities established from BFT play a crucial role as natural bioremediation agents, maintaining water quality and converting nitrogenous waste (2). They also contribute to the development of nutrient-rich flocs that serve as food sources, supporting high-density aquaculture growth. Studies characterizing BFT microbiota have identified complex bacterial populations, predominantly *Proteobacteria* and *Bacteroidetes* (21, 22). BFT promotes the growth of both autotrophic and heterotrophic microbes (23, 24), including beneficial communities like *Bacillus*, *Acinetobacter*, *Sphingomonas*, *Pseudomonas*, *Rhodopseudomonas*, *Micrococcus*, *Nitrosomonas*, *Nitrospira*, *Nitrobacter*, *Cellulomonas*, and yeast (2). However, more research is needed on the microbial species or diversity responsible for developing BFT and maintaining optimal water quality and health of cultured animals.

The Japanese eel (*Anguilla japonica*), one of 16 species of freshwater eel in the *Anguilla* genus, is naturally found in the freshwater of East Asia and commonly cultured and consumed as a luxury food item in China, Japan, and South Korea (25, 26). The lifecycle of the Japanese eel remains largely unknown, with recent discoveries of its spawning area (27). Complete aquaculture is not feasible, so young eels are captured from the wild and grown in aquaculture facilities. Prior to the research by Hwang et al., there had been no studies comparing the growth of Japanese eel using BFT. This study reported that increased BFT treatment correlates with higher growth rates in eels (28). Follow-up studies with European eel by Vinatea et al. supported these findings and indicated that production costs using BFT are lower than conventional methods (29). However, the direct impact of BFT on eel growth factors, especially gut microbiome and water microbiome changes, is not well understood.

In this study, we observed the survival and growth rates of eels treated with BFT over 8 weeks and monitored changes in the gut and water microbiome. FTS served as a negative control, and microbiome community was investigated using 16S rRNA amplicon sequencing and quantitative PCR (qPCR). We investigated how BFT treatment affects water quality and various aspects of eel growth physiology, focusing on changes in the alpha and beta diversity of microbiome. We tracked which microbial residents showed significant changes in abundance due to BFT treatment. Among these, we identified key candidates that significantly influence eel growth rates using random forest analysis. Our research highlights how BFT in aquaculture links food productivity with microbiome changes.

## MATERIALS AND METHODS

### Establishing the ecological framework for eel growth in BFT and FTS treatments

The aquaculture experiments took place in a 0.5-ton polypropylene circular tank (50 cm × 50 cm). Two rearing conditions were assessed: the BFT and FTS treatments, each with three replicates. The two treatments received water at a rate of 100 mL per minute, ensuring a daily turnover rate of 53.3%. BFT tanks were inoculated with 10 ppm of live bacteria specific to BFT (*Bacillus amyloliquefaciens*, *Bacillus licheniformis*, *Bacillus subtilis*, *Cellulomonas denitrificans*, *Cellulomonas* sp., *Nitrobacter winogradskyi*, *Nitrosomonas europaea*, *Pseudomonas stutzeri*, and *Rhodopseudomonas palustris*) (BFT-ST, EgeeTech Ltd., USA). The carbon-to-nitrogen (C:N) ratio in BFT water followed a previous method (30, 31). To achieve a 15:1 C:N ratio, 10 g of molasses was added daily with 30 g of feed as a nitrogen source.

The Japanese eels used in the experiment were acquired from a general aquaculture store during the glass eel season and randomly selected from those housed at the Advanced Aquaculture Research Center (Jinhae, South Korea). The experiment included three tanks for each system: BFT (*n* = 31 + 28 + 27) and FTS (*n* = 30 + 29 + 27). Eels, with an average weight of 175.6 ± 10.73 g (BFT) and 173.3 ± 9.12 g (FTS), were allocated to each tank with a total weight of 5.0 ± 0.08 kg (BFT) and 5.0 ± 0.07 kg (FTS), resulting in a density of 9.98 ± 0.15 kg/m³ per tank. The experiment commenced after a 1-week acclimation period to ensure the eels adjusted to the tanks. Throughout the study, the eels were fed a commercial feed “Gold-Eel” (containing 52% crude protein, 12% crude fat, 1.0% Ca, 12% moisture, 2.7% P, 6.0% crude fiber, and 18% crude ash) produced by Cargill Agri Purina (Seongnam, South Korea) at a rate of 1% of their total weight per day. The water temperature was maintained at 25 ± 1°C using a 1 kW heater (OKE-HE-100, Sewon OKE, Busan, Korea), and dissolved oxygen (DO) levels were kept above 7 mg/L. Biofloc suspension was ensured by aerating each tank with two air stones, and an additional high-pressure diffuser was installed in the BFT tank to maintain DO levels.

Water samples were collected at a time point 8 weeks after the start of aquaculture, with three replicates per tank (a total of nine replicates). Water quality parameters, including temperature, DO, and pH, were measured at sampling points (A, B, C, and D; see Fig. S1) before the morning feeding using a multi-parameter water quality meter (YSI-650; Yellow Springs Instruments, Yellow Springs, Ohio, USA). Total ammonia nitrogen (TAN) and nitrite nitrogen (NO₂⁻-N) levels were also measured prior to feeding and analyzed using colorimetric methods with Spectroquant Ammonium and Nitrite Test kits (Merck KGaA, Darmstadt, Germany) via a Ultraviolet/Visible spectrophotometer Prove 600 plus (Merck KGaA, Darmstadt, Germany).

### Molecular experiments

Triplicate 500 mL water samples were collected from each treatment and filtered through a 0.22 µm pore size membrane filter (Hyundai Micro, Seoul, South Korea) using a Vacuum Membrane Filter Holder (AccuResearch Korea Inc., Seoul, South Korea). Genomic DNA was extracted from the filtered samples using a PowerSoil DNA extraction kit (MoBio, Carlsbad, CA, USA) according to the manufacturer’s protocol.

For microbial metabarcoding, bacterial 16S rRNA regions were amplified with primers 515F and 806R (32) and Illumina adaptors. PCR amplifications were performed using a SimpliAmp Thermal Cycler (Thermo Fisher Scientific, MA, USA) with AccuPower PCR premix (Bioneer, Daejeon, South Korea), following these conditions: initial denaturation at 94°C for 5 minutes, 35 cycles of denaturation at 94°C for 30 seconds, annealing at 55°C for 30 seconds, extension at 72°C for 40 seconds, and a final extension at 72°C for 10 minutes. The final PCR reaction volume was 20 µL, containing 10 pmol of each primer and 2 µL of genomic DNA. PCR products were evaluated using gel electrophoresis on a 1% agarose gel and purified with the Expin PCR Purification Kit (GeneAll Biotechnology, Seoul, South Korea). To minimize stochastic PCR biases, each sample was replicated three times and combined into a single pool.

qPCR was used to determine the total bacterial load, targeting the rRNA gene for overall bacteria (V4–V5 region). The qPCR analysis was conducted using Luna Universal qPCR Master Mix (New England Biolabs, Frankfurt, Germany), a QuantStudio 6 pro Real-Time PCR system (Thermo Fisher Scientific), and QuantStudio Design & Analysis Software v1.5.2 (Thermo Fisher Scientific). This analysis was supported by the Gyeongnam Bio and Anti-aging Core Facility Center (Changwon National University, South Korea).

### Bioinformatics and statistics

Sequencing of the amplicon library was performed on an Illumina MiSeq platform at Macrogen (Seoul, South Korea). The raw data were processed using QIIME2 (33), including demultiplexing and denoising via the DADA2 pipeline (34). Taxonomic assignment of microbial sequences was carried out using the VSEARCH protocol (35), aligned against the database SILVA (36) with a 97% similarity threshold. Phylogenetic analysis was conducted using the q2-alignment plugin with RAxML (37). The sequence data were deposited in the National Center for Biotechnology Information Sequence Read Archive under accession number PRJNA1154397 (Table S1). The operational taxonomic unit (OTU) table was imported from QIIME2 into R using the “qiime2R” package (https://github.com/jbisanz/qiime2R/) and then rarefied to 11,000 sequence reads per sample using “phyloseq” (38), excluding a single BFT-treated gut sample (Bg3_3; accession number SAMN43414841; approximately 6,000 reads). Subsequent analyses were based on this rarefied OTU table.

All statistical analyses and visualizations were performed using R v.3.5 (39). Normality of the data was assessed with the Shapiro-Wilk test (40), and non-parametric tests were employed due to violations of normality assumptions. These included the Kruskal-Wallis test (41) with Bonferroni correction (42) for multiple comparisons and the Mann-Whitney U test (43) for pairwise comparisons. Figures were created using the “ggplot2” and “ggpubr” packages (44, 45). The survival rate of eels was analyzed using the “survival” and “survminer” packages (46). Microbiome rarefaction analysis was conducted with the “iNEXT” package (47). Shared OTUs among microbiome were analyzed using the “UpsetR” package (48), while nestedness and partitioning variable analyses were performed with the “vegan” package (49). Hierarchical heatmaps were visualized using “pheatmap” (50). Non-metric multidimensional scaling (NMDS) was used with the “vegan” package to visualize the similarity of water properties and bacterial communities. Beta diversity of the microbiome was calculated based on UniFrac distances (51). The sample distances from beta diversity (UniFrac) were analyzed using the permutational analysis of variance (PERMANOVA) test, performed with the “betatest” function in R. Sankey diagrams were created using “http://sankeymatic.com.” Differentially abundant OTUs were visualized using volcano plots (52) based on chi-squared tests (53). Random forest analyses were conducted with the “randomForest” and “rfUtilities” packages, using 501 decision trees and 1,000 permutations (54).

## RESULTS

### BFT treatment was more advantageous for eel growth compared with FTS treatment

**Fig 1:**
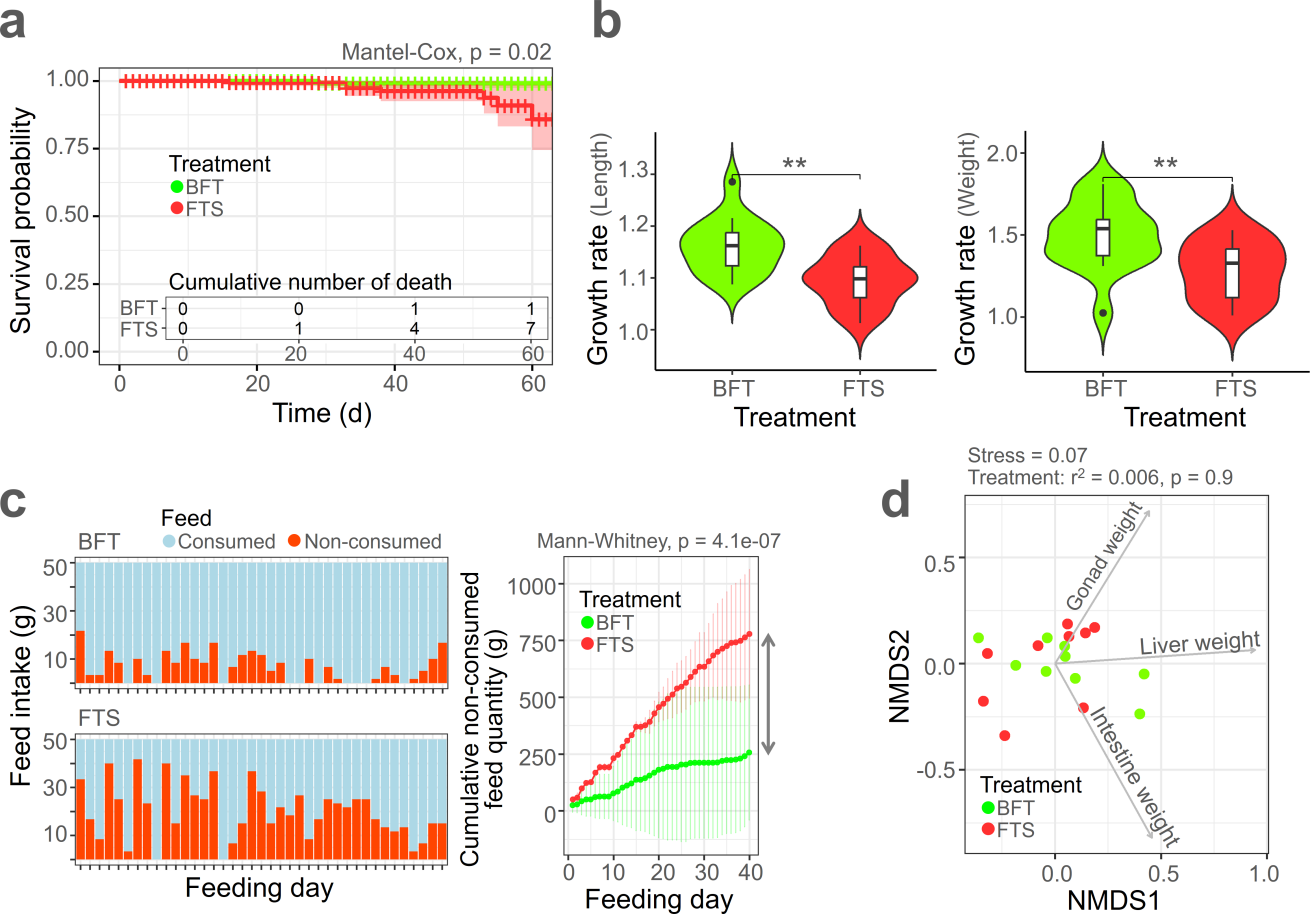
Physiology profile of eel growth. (**a**) Survival rate of eels between the two treatments, with cumulative mortality recorded. (**b**) Relative growth rates in length and weight of eels between the two treatments. (**c**) Consumed feed rate of eels between the two treatments. (**d**) NMDS plot of eel internal organ weights based on Bray-Curtis dissimilarity. **P < 0.01.

A significant difference in eel survival rate was observed between the two treatment groups (Mantel-Cox, *P* = 0.02), with the BFT-treated group exhibiting a higher survival rate than the FTS-treated group (Fig. 1a). During the 60-day experimental period, only one eel died in the BFT-treated group, whereas six eels died in the FTS-treated group. In the three tanks of each system, one eel died in one of the BFT tanks, while in the FTS tanks, three eels died in one tank, two eels in another, and one eel in the third. A significant difference in growth rate was noted between the BFT and FTS treatments (Fig. 1b), with the BFT treatment resulting in higher growth rates for both length (*P* < 0.01) and weight (*P* < 0.01) of the eels. The feed consumption rate showed substantial differences between the two treatment groups (Fig. 1c), with the FTS-treated group exhibiting a higher non-consumed feed rate throughout the feeding days. When comparing the cumulative non-consumed feed quantity, a statistically significant difference was found between the two groups (*P* = 4.1e−07). No significant differences were observed in the weights of the gonad, liver, and intestine between the BFT and FTS treatment groups (Fig. 1d, *r*² = 0.006, *P* = 0.9). Water quality parameters between the BFT and FTS treatment groups showed minor differences (Fig. S1a, *r*² =0.91, *P* = 0.1). DO, pH, and NO_2_^−^-N initially exhibited slight differences between the BFT and FTS treatments at the first sampling point. However, as time progressed, these differences diminished, and there were no significant disparities between the two treatments. Additionally, temperature remained similar and stable between the two groups throughout the study (Fig. S1b through f).

### Alpha diversity differed between BFT and FTS treatments in water microbiome, not in the gut

**Fig 2:**
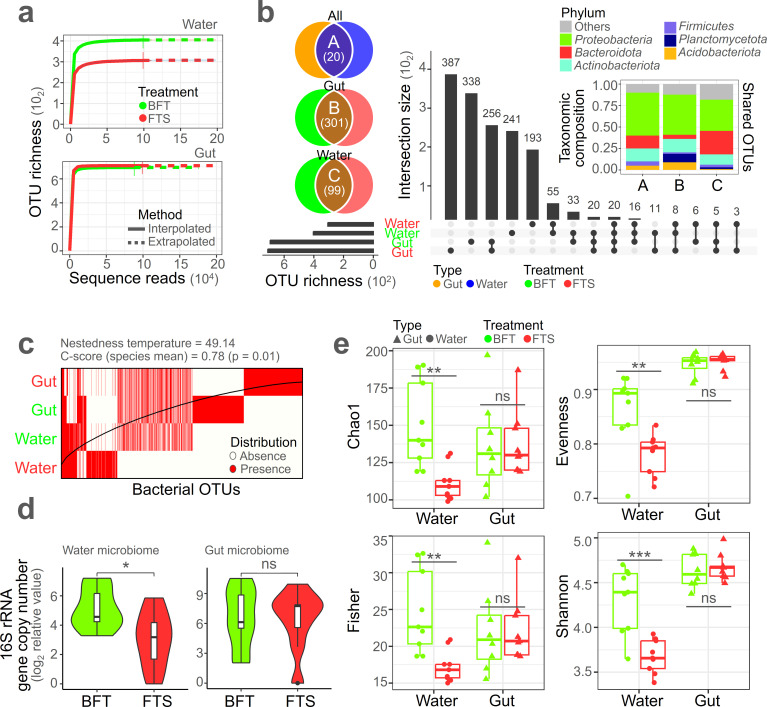
Alpha diversity of eel gut and water microbiome. (**A**) Rarefaction curves of microbiome between the two treatments. (**B**) Shared OTU profile of microbiome for each condition. The stacked bar plot shows the taxonomic composition of shared OTUs based on simple OTU composition, not relative abundance. (**C**) Nestedness analysis of microbiome for each condition. (**D**) qPCR results of microbiome for each condition (general bacterial amount: 16S rRNA gene copy number). (**E**) Alpha diversity of microbiome. ***P < 0.005, **P < 0.01, and *P < 0.05; ns, not significant.

A rarefaction test was conducted to determine if the sequence reads covered sufficient OTU richness (Fig. 2a). The rarefied curve indicated that the sampling effort in this study was adequate to explore OTU diversity. While the sequence reads in water and gut microbiome showed OTU richness saturation before reaching 10,000 reads, each condition in this study used approximately 100,000 sequence reads. Notably, the water microbiome showed higher saturated richness in the BFT treatment compared with the FTS treatment, but there was no significant difference of OTU richness in the gut microbiome between the two conditions.

An investigation was conducted to identify which OTUs were shared among the four conditions (BFT water, FTS water, BFT gut, and FTS gut microbiome) (Fig. 2b). Interestingly, the gut and water microbiome shared only 20 OTUs (group A). Between the BFT and FTS treatments, the gut microbiome shared 301 OTUs (group B), and the water microbiome shared 99 OTUs (group C), suggesting that the gut microbiome are more similar to each other than the water microbiome. The taxonomic composition of the shared OTUs revealed that *Proteobacteria* predominated across groups A, B, and C, while *Bacteroidota* were relatively less abundant in group B than in C, and *Planctomyceta* were more abundant in B than in C.

Analysis of the overall nestedness of the microbiome indicated that the gut microbiome had a more nested structure compared with the water microbiome (Fig. 2c, nestedness temperature = 49.14, C-score = 0.78, *P* = 0.01). OTU diversity was higher in the FTS treatment than in the BFT treatment for the gut microbiome, while the opposite trend was observed for the water microbiome. The qPCR results indicate a significant difference in the 16S rRNA gene copy number in the water microbiome between the BFT and FTS treatments, with higher values observed in the BFT treatment (Fig. 2d, Mann-Whitney, *P* < 0.05). However, the gut microbiome did not show a statistically significant difference in the quantity of bacterial community between the two treatments, with the median value being higher in FTS compared with BFT. For alpha diversity, all four indices (Chao1, Evenness, Fisher, and Shannon) showed higher values in the BFT-treated water microbiome compared with the FTS-treated water microbiome (Mann-Whitney, *P* < 0.01), but no significant difference was observed between the two treatments in the gut microbiome (Fig. 2e).

### Water microbiome community structures differed between BFT and FTS treatments, but gut microbiome did not

**Fig 3:**
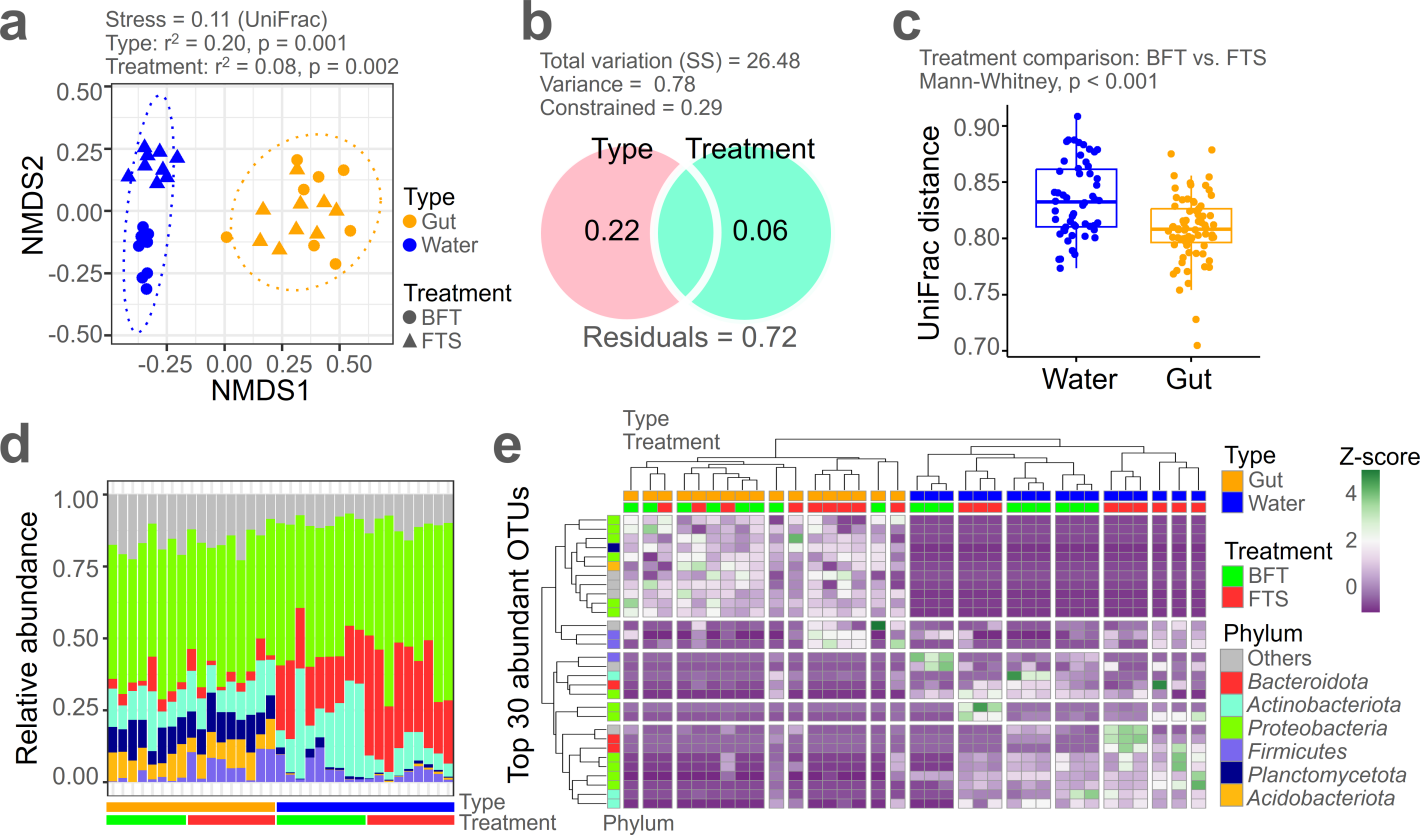
Beta diversity of eel gut and water microbiome. (**a**) NMDS results of the overall microbiome. (**b**) Partitioning variables results to determine the impact of type and treatment on community structure. (**c**) Comparison of Unifrac distances between gut and water conditions for BFT and FTS treatments, indicating community dissimilarity. (**d**) Phylum-level taxonomic composition of the microbiome. (**e**) Hierarchical heatmap of the top 30 abundant OTUs in the overall microbiome.

NMDS ordination clearly distinguished between gut and water microbiome (Fig. 3a, type: *r*² = 0.20, *P* = 0.001) and also showed statistically significant differences between treatments (BFT and FTS) (treatment: *r*² = 0.08, *P* = 0.002). Partitioning variables analysis revealed that the type of microbiome (proportion = 0.22) had a greater influence on community structure than the treatment (0.06), as shown in Fig. 3b. In the NMDS plot, the gut microbiome did not show pronounced community structure differences between BFT and FTS treatments, as supported by the comparison of UniFrac distances between BFT and FTS gut microbiome (Fig. 3c) and NMDS analysis of water and gut microbiome separately (Fig. S2a). Additionally, the intra-community distance was larger in the BFT-treated gut microbiome compared with the FTS-treated gut microbiome (Mann-Whitney, *P* < 0.001), whereas there was no significant variance difference between treatments in the water microbiome (Fig. S2b). In summary, while the community structure of the microbiome in the water significantly differs between the two treatment groups, the gut microbiome does not show such substantial differences.

At the phylum level, *Proteobacteria* predominated across both microbiome. The gut microbiome had relatively higher proportions of *Planctomyceta* and *Acidobacteriota*, while the water microbiome had a relatively higher proportion of *Bacteroidota* (Fig. 3d). At the order level, no major differences were found between BFT and FTS treatments in the gut microbiome, but in the water microbiome, the FTS treatment had a relatively higher proportion of *Flavobacteriales* (Fig. S2c). The distribution of the top 30 abundant OTUs clearly clustered the gut and water microbiome hierarchically (Fig. 3e). Within each microbiome, the water microbiome was distinctly separated by treatment, unlike the gut microbiome. The profiles of top 30 abundant OTUs, including OTU0674: f__*Weeksellaceae*, OTU0324: g__hgcI_clade, and OTU0373: g__*Flectobacillus*, are provided in Fig. S2d. NMDS1 scores significantly differentiated gut and water microbiome (Fig. 3a).

Random forest analysis identified the top 10 informative OTUs contributing most to the NMDS1 score (Fig. S4a, *r*² = 0.91, *P* = 0.001), revealing OTUs abundant in the water microbiome, such as OTU1529: g__*Acinetobacter* and OTU0394: g__*Polynucleobacter*, and those frequent in the gut microbiome, such as OTU0721: g__*Sphingomonas* and OTU0131: f__*Pleomorphomonadaceae*. Additionally, NMDS2 scores distinguished BFT and FTS treatments within the water microbiome (Fig. S4b, *r*^2^ = 0.85, *P* = 0.001). Random forest analysis showed that OTU0864: *Coraliomargarita* sp. and OTU1102: f__*Chitinophagaceae* were abundant in the FTS-treated water microbiome, while OTU1053: g__*Duganella* and OTU0138: f__*Micropepsaceae* were abundant in the BFT-treated water microbiome (Fig. S3b).

### Random forest analysis identified bacterial OTUs considered to influence eel growth

**Fig 4:**
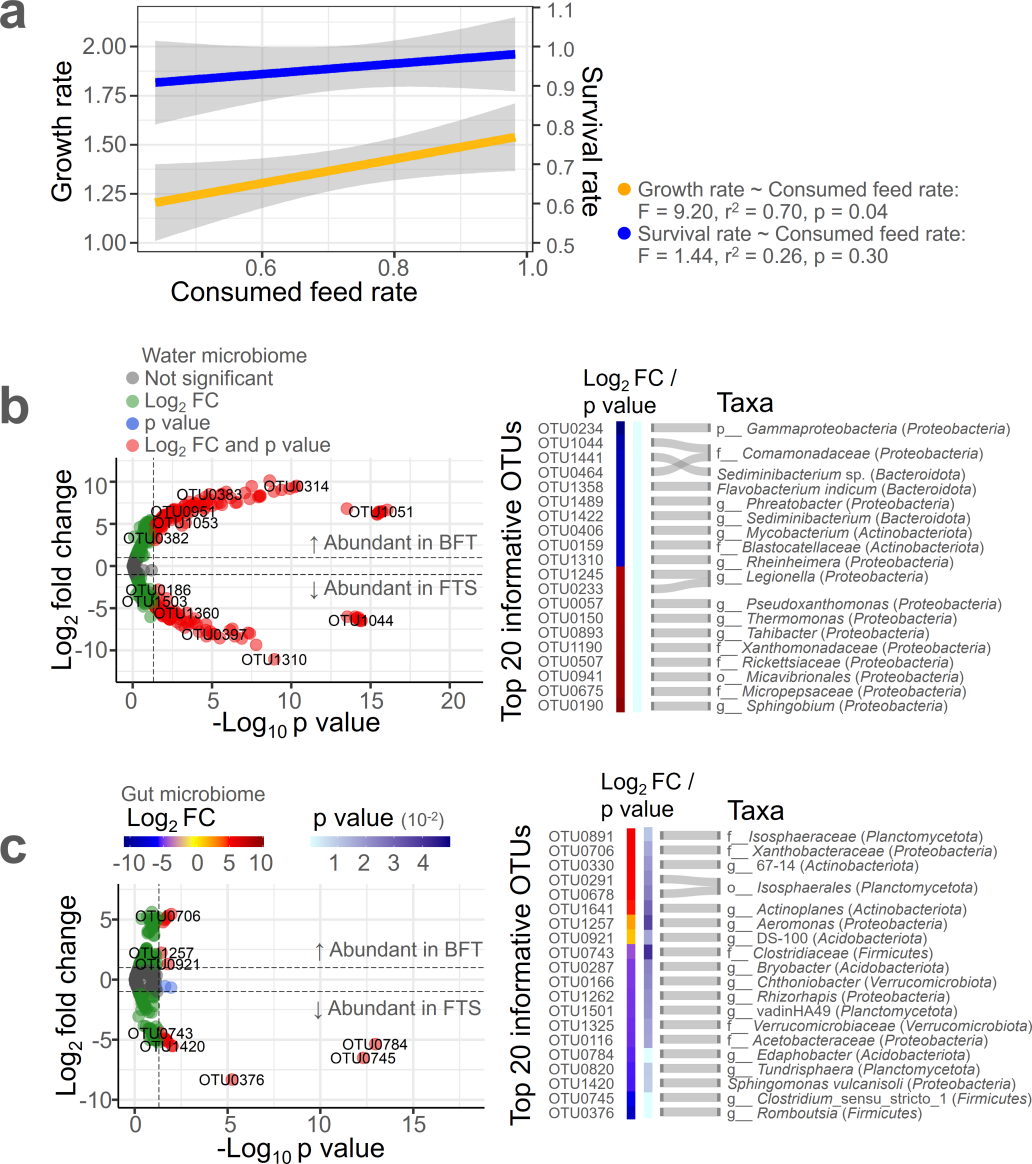
Identification of bacterial residents most affecting eel growth physiology. (**a**) Regression analysis showing the correlation between consumed feed rate, growth rate, and survival rate of eels. Profiles of the most differentially abundant OTUs between BFT and FTS treatments in water (**b**) and gut (**c**), determined by chi-squared tests.

The growth of eels exhibited significant differences between the BFT and FTS treatments (Fig. 1). This variation is likely associated with the consumed feed rate, as determined through regression analysis, which demonstrated the impact of consumed feed rate on growth rate and survival rate (Fig. 4a). An increase in consumed feed rate correlated with an increase in survival rate (*r*² = 0.26, *P* = 0.30) and growth rate (*r*² = 0.70, *P* = 0.04).

To identify the bacterial OTUs influencing the consumed feed rate of eels, differential abundant OTUs in the water microbiome between BFT and FTS treatments were identified using chi-squared tests (Fig. 4b). The top 20 informative OTUs, based on the absolute value of Log_2_ FC, were identified. Notably, OTU0190: g__*Sphingobium* and OTU0675: f__*Micropepsaceae* had higher relative abundance in the BFT environment, while OTU0234: p__*Gammaproteobacteria* and OTU1044: f__*Comamonadaceae* were more abundant in the FTS environment. Similarly, differential abundant OTUs in the gut microbiome for both BFT and FTS environments were identified (Fig. 4c). Among the top 20 informative OTUs from both the water and gut microbiome (totaling 40 OTUs), the OTUs most significantly impacting the eel consumed feed rate were identified using random forest analysis (Fig. S4). In the water microbiome (*r*² = 0.61, *P* = 0.001), OTU1310: g__*Rheinheimera* from the FTS environment had the greatest impact on the consumed feed rate, while in the gut microbiome (*r*² = 0.23, *P* = 0.013), OTU0376: g__*Romboutsia* from the FTS environment was the most influential.

## DISCUSSION

Since the applicability of the BFT to freshwater fish was reported (9), studies on the growth of major inland fish species such as catfish, tilapia, and carp have been documented (37, 55, 56). However, research on the application of BFT to Japanese eel, which is considered a delicacy in East Asia, is relatively scarce. Our study found that feed conversion, growth, and survival rates of eels were all higher in the BFT treatment compared with the FTS. Our findings that BFT is advantageous for eel growth align with previous studies (28, 29). High nitrate concentrations in eel farming are known to affect water quality and reduce bacterial populations (28, 57). However, in this study, the BFT did not show lower nitrogen waste level compared with the negative control FTS. This suggests that factors other than nitrogen might have influenced eel growth. Microbial communities associated with BFT have been suggested to contribute positively to fish health by potentially supporting immune function and limiting the proliferation of harmful bacteria (58, 59). Moreover, microorganisms in BFT may play roles in producing biomolecules, such as enzymes and bioactive compounds, that could hold value in aquaculture (20). Further research is needed to explore the functionally diverse aspects of the changing microbiome following BFT treatment.

The higher alpha diversity and bacterial community abundance (16S rRNA gene copy number) in the BFT water microbiome compared with the FTS appear reasonable, given that BFT is known to enhance microbial quantities (19, 37). In terms of beta diversity, two noteworthy observations were made: the distinct differences between the gut and water microbiome and the significant divergence between the BFT and FTS treatments within the water microbiome. While some of these observations are present, further research is needed to clarify their implications. Specifically, the differences in the microbiomes between the gut and water, as well as the divergence between the BFT and FTS treatments, were observed. The random forest analysis identified certain genera like *Acinetobacter* and *Polynucleobacter* as more prevalent in the water microbiome and *Sphingomonas* and *Pleomorphomonadaceae* in the gut microbiome. These patterns suggest potential influences on community structure but do not prove causality. For instance, the reduced presence of *Acinetobacter* in the gut suggests the eels are relatively healthy, and *Polynucleobacter’s* role in biological waste treatment points to efficient water management. *Sphingomonas*, known for its positive effects on fish health, was more dominant in the gut, suggesting potential beneficial effects. Further studies are needed to explore the functional roles of these genera and to understand why certain OTUs, like *Coraliomargarita* and *Duganella*, were abundant in specific treatments. Additionally, the link between certain taxa and eel feed consumption rates, observed in the FTS treatment, warrants further functional investigation to understand their potential impacts on eel growth and health.

Our study has a limitation, specifically, while we examined how OTU distribution varied across treatments, we did not determine the functional roles these OTUs play within the given ecosystem. Nevertheless, our study serves primarily as a preliminary report, highlighting the need for further functional research on each OTU. An effective follow-up study could involve isolating these OTUs or species using culture-dependent methods, testing their extracts, and examining their effects on fish growth physiology. Despite these limitations, our research has illuminated the differences in bacterial community structure between BFT and FTS treatments in the relatively understudied Japanese eel-associated microbiome, examining both water and gut microbiome. In aquaculture, traditional metrics such as nutrient cycling and toxic substance levels have been the focus of studies on fish growth. However, given the current understanding of the significant impact of microbiome on hosts and their environments, it is now time to also consider other detailed factors. This foundational data will be valuable for future metagenomics-based aquaculture studies.

## Data Availability

Sequencing data were deposited in the National Center for Biotechnology Information Sequence Read Archive under accession number PRJNA1154397.

## SUPPLEMENTAL MATERIAL

Supplemental material spectrum.02206-24-s0001.docx Fig. S1 to S4; Table S1.

ASM does not own the copyrights to Supplemental Material that may be linked to, or accessed through, an article. The authors have granted ASM a non-exclusive, world-wide license to publish the Supplemental Material files. Please contact the corresponding author directly for reuse.
