# Supplementary material for: Biofloc technology significantly reshapes water microbiome and improves survival rates in Japanese eel (Anguilla japonica)
Source: Microbiol Spectr. 2025 Jan 15;13(2):e02206-24. doi: 10.1128/spectrum.02206-24 (PMC11792490; doi:10.1128/spectrum.02206-24)
Supplement: Supplemental material — Fig. S1 to S4; Table S1. [file spectrum.02206-24-s0001.docx]

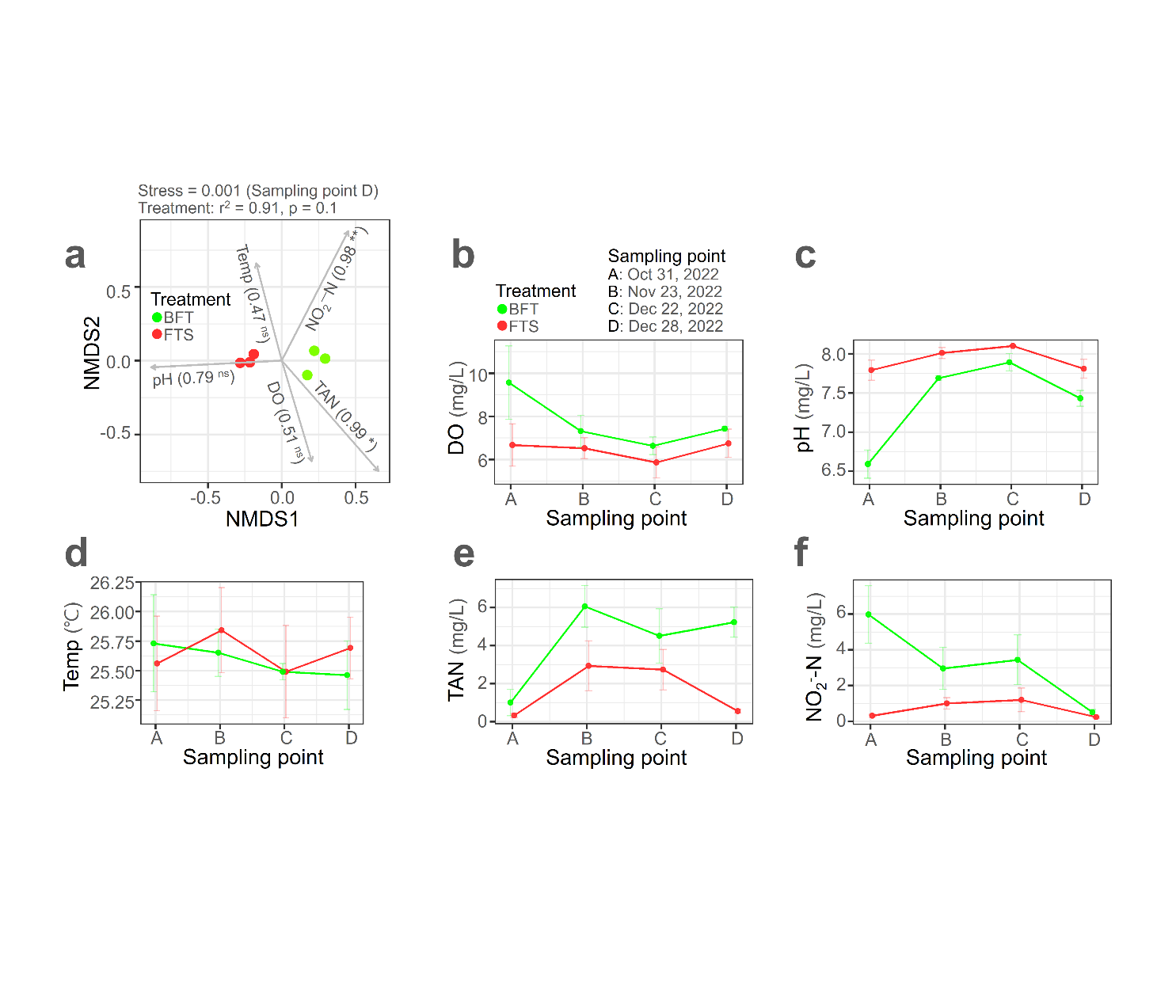


**Figure S1. Comparison of water property differences under varying treatments**

Five water properties were visualized using NMDS to illustrate overall patterns of variation based on treatment (a). Temporal changes in DO (b), pH (c), water temperature (d), TAN (e), and NO_2_ (f) were represented using line graphs with error bars indicating standard deviation.


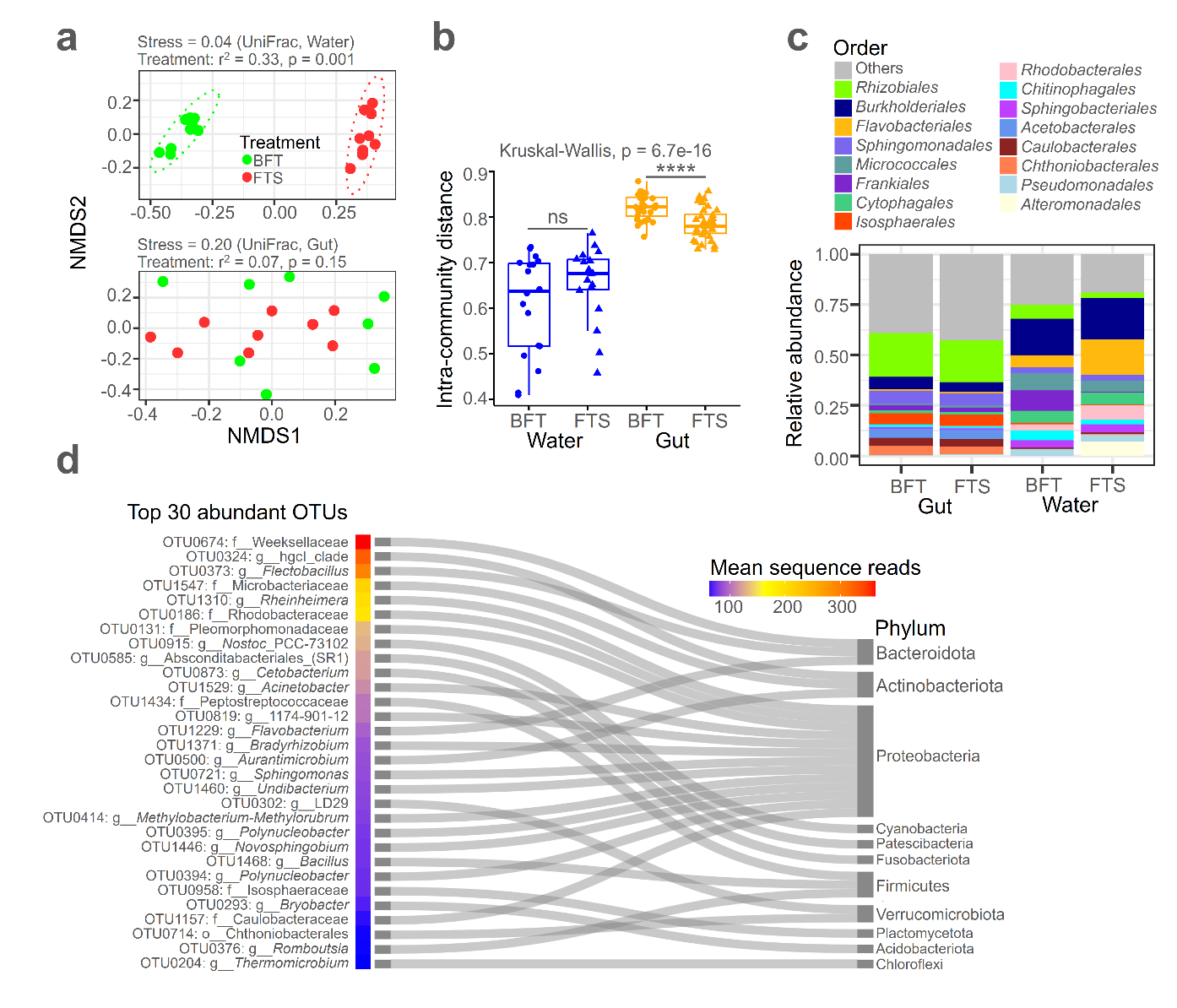


**Figure S2. Supplementary analysis of beta diversity in water and gut microbiomes**

To minimize the masking of treatment-specific differences, water and gut microbiomes were separately ordinated (a). Variations in intra-community distances within each microbiome type were displayed as boxplots (b). Taxonomic composition at the order level was presented (c), along with profiles of the 30 most abundant OTUs (d).


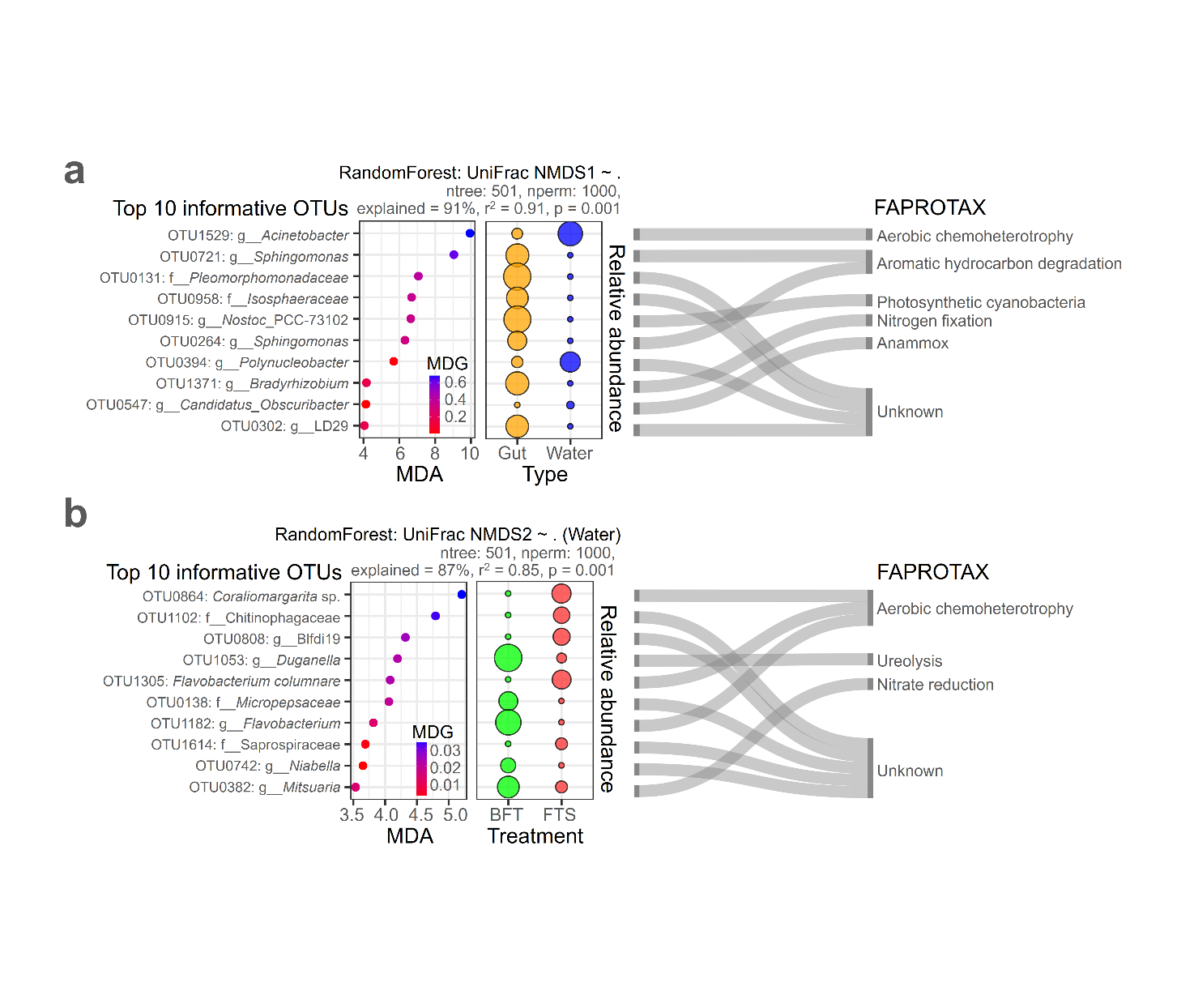


**Figure S3. Informative OTUs strongly influencing NMDS scores**

The top 10 OTUs contributing most significantly to NMDS1 variation across the entire microbiome (a) and NMDS2 variation within the water microbiome (b) were identified and listed.


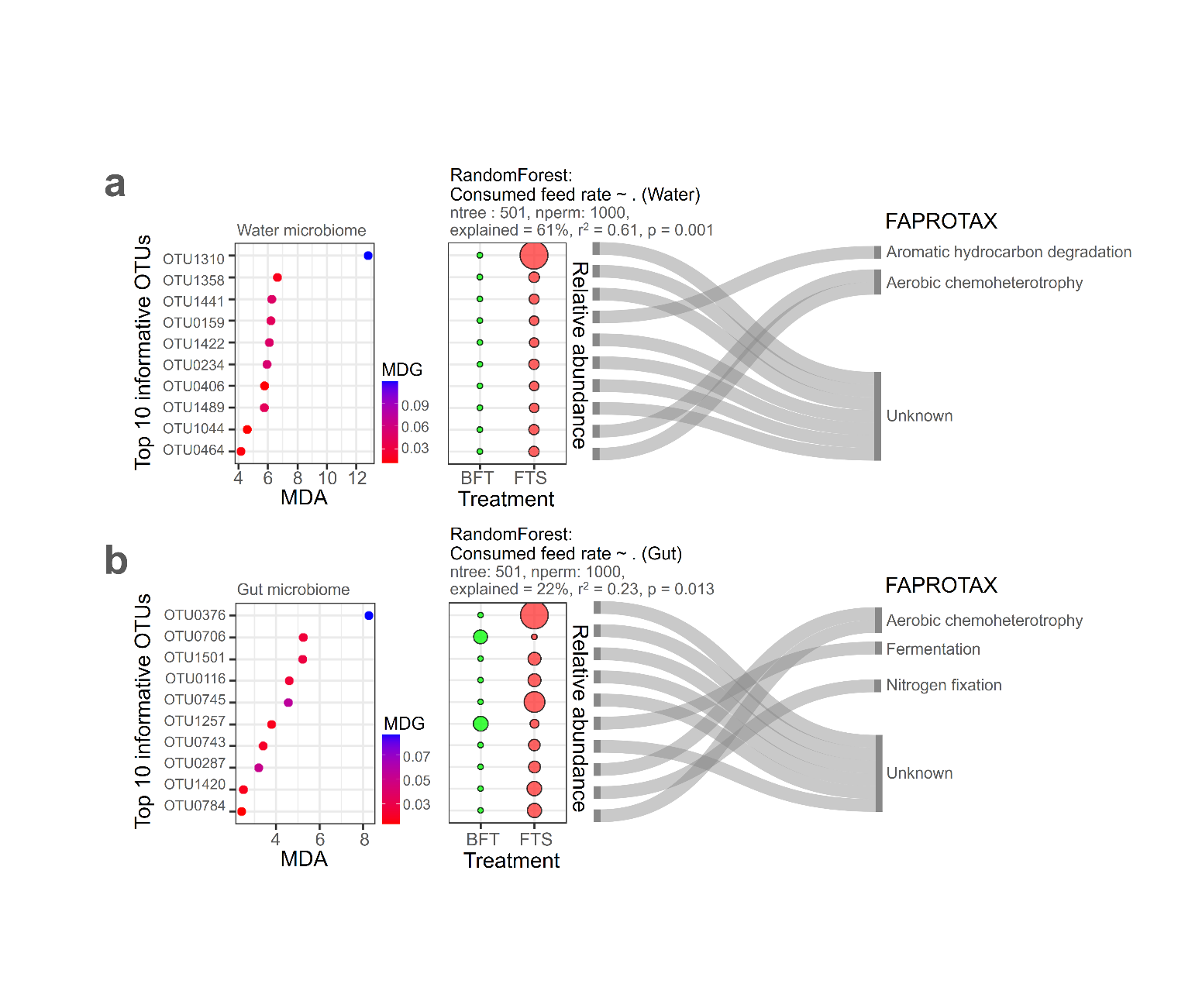


**Figure S4. Informative OTUs significantly impacting feed consumption rates in eels**

OTUs from the water microbiome (a) and gut microbiome (b) identified as the top 10 contributors to variations in feed consumption rates were highlighted.

**Table S1. Bioinformation of sample derived from amplicon sequencing in this study**

| Accession no. | Sample name | Type | Treatment | Tank |
| --- | --- | --- | --- | --- |
| SRR30524524 | Bg1_1 | gut | BFT | a |
| SRR30524523 | Bg1_2 | gut | BFT | a |
| SRR30524512 | Bg1_3 | gut | BFT | a |
| SRR30524501 | Bg2_1 | gut | BFT | b |
| SRR30524494 | Bg2_2 | gut | BFT | b |
| SRR30524493 | Bg2_3 | gut | BFT | b |
| SRR30524492 | Bg3_1 | gut | BFT | c |
| SRR30524491 | Bg3_2 | gut | BFT | c |
| SRR30524490 | Bg3_3 | gut | BFT | c |
| SRR30524489 | Cg1_1 | gut | FTS | d |
| SRR30524522 | Cg1_2 | gut | FTS | d |
| SRR30524521 | Cg1_3 | gut | FTS | d |
| SRR30524520 | Cg2_1 | gut | FTS | e |
| SRR30524519 | Cg2_2 | gut | FTS | e |
| SRR30524518 | Cg2_3 | gut | FTS | e |
| SRR30524517 | Cg3_1 | gut | FTS | f |
| SRR30524516 | Cg3_2 | gut | FTS | f |
| SRR30524515 | Cg3_3 | gut | FTS | f |
| SRR30524514 | W8B1 | water | BFT | a |
| SRR30524513 | W8B2 | water | BFT | b |
| SRR30524511 | W8B3 | water | BFT | c |
| SRR30524510 | W8C1 | water | FTS | d |
| SRR30524509 | W8C2 | water | FTS | e |
| SRR30524508 | W8C3 | water | FTS | f |
| SRR30524507 | 8WB11 | water | BFT | a |
| SRR30524506 | 8WB12 | water | BFT | a |
| SRR30524505 | 8WB21 | water | BFT | b |
| SRR30524504 | 8WB22 | water | BFT | b |
| SRR30524503 | 8WB31 | water | BFT | c |
| SRR30524502 | 8WB32 | water | BFT | c |
| SRR30524500 | 8WC11 | water | FTS | d |
| SRR30524499 | 8WC12 | water | FTS | d |
| SRR30524498 | 8WC21 | water | FTS | e |
| SRR30524497 | 8WC22 | water | FTS | e |
| SRR30524496 | 8WC31 | water | FTS | f |
| SRR30524495 | 8WC32 | water | FTS | f |
